# Assessment of immunoprecipitation with subsequent immunoassays for the blood-based diagnosis of Alzheimer’s disease

**DOI:** 10.1007/s00406-023-01751-2

**Published:** 2024-02-06

**Authors:** Barbara Morgado, Hans-Wolfgang Klafki, Chris Bauer, Katharina Waniek, Hermann Esselmann, Oliver Wirths, Niels Hansen, Ingolf Lachmann, Dirk Osterloh, Johannes Schuchhardt, Jens Wiltfang

**Affiliations:** 1 Department of Psychiatry and Psychotherapy, University Medical Center Goettingen (UMG), Georg-August University, Von-Siebold Strasse 5, 37075 Goettingen, Germany; 2 MicroDiscovery GmbH, Marienburger Strasse 1, 10405 Berlin, Germany; 3 Roboscreen GmbH, Hohmannstrasse 7, 04129 Leipzig, Germany; 4 German Center for Neurodegenerative Diseases (DZNE), 37075 Goettingen, Germany; 5 Neurosciences and Signaling Group, Institute of Biomedicine (iBiMED), Department of Medical Sciences, University of Aveiro, 3810-193 Aveiro, Portugal

**Keywords:** Alzheimer’s disease, Amyloid beta, Tau, Blood plasma, Biomarker

## Abstract

The Aβ42/40 ratio and the concentration of phosphorylated Tau181 in blood plasma represent attractive biomarkers for Alzheimer's disease. As a means for reducing potential matrix effects, which may interfere with plasma immunoassays, we have previously developed a pre-analytical sample workup by semi-automated immunoprecipitation. Here we test the compatibility of pre-analytical immunoprecipitations with automated Aβ1-40, Aβ1-42 and phosphorylated Tau181 immunoassays on the Lumipulse platform and compare the diagnostic performance of the respective immunoprecipitation immunoassay approaches with direct plasma measurements. 71 participants were dichotomized according to their Aβ42/40 ratios in cerebrospinal fluid into the diagnostic groups amyloid-positive (*n = *32) and amyloid-negative (*n = *39). The plasma Aβ1-42/1-40 ratio and phosphorylated Tau181 levels were determined on the Lumipulse G600II platform (Fujirebio) by direct measurements in EDTA–plasma or after Aβ- or Tau-immunoprecipitation, respectively. Pre-analytical immunoprecipitation of Aβ turned out to be compatible with the Lumipulse Aβ assays and resulted in a numerical, yet statistically not significant increase in the area under the ROC curve for plasma Aβ1-42/1-40. Additionally, we observed a significant increase in the standardised effect size (Cohen’s D). Pre-analytical immunoprecipitation of Tau resulted in increased differences between the diagnostic groups in terms of median and mean phosphorylated Tau 181 levels. Furthermore, we observed a greater Cohen’s d (*p < *0.001) and a larger area under the ROC curve (*p = *0.038) after Tau-IP. Our preliminary findings in a small, preselected sample indicate that pre-analytical immunoprecipitation may have the potential to improve the diagnostic performance of plasma biomarker immunoassays for Aβ1-42/1-40 and phosphorylated Tau181 to predict brain amyloid deposition.

## Introduction

Alzheimer’s disease (AD) is the most common cause of dementia [1]. Preclinical AD is characterised by early molecular changes in the brain in the absence of overt and severe cognitive symptoms. The typical pathophysiological changes found in AD brains include accumulation of extracellular amyloid plaques and intraneuronal neurofibrillary tangles, composed of aggregated amyloid-β (Aβ) peptides and hyperphosphorylated Tau protein (pTau), respectively [2, 3]. Clinical AD (dementia due to AD) is diagnosed at a relative late stage after substantial irreversible cognitive deficits have manifested. Early, pre-dementia diagnosis based on biomarkers has become increasingly important, in particular regarding the development of novel disease-modifying treatments [4], which need to be initiated in an early disease stage to be most effective.

In 2016, a framework was proposed that grouped AD biomarkers into three categories: biomarkers of amyloid pathology (A), hyperphosphorylated Tau aggregates (T) and neurodegeneration (N) [5]. The classification of study participants according to the AT(N) scheme can be made on the basis of biomarker measurements in cerebrospinal fluid (CSF (CSF Aβ42, CSF Aβ42/40, phospho-Tau, total Tau) and/or imaging (amyloid positron emission tomography (PET), Tau-PET, structural MRI and FDG-PET) [6, 7]. Efforts have been made to explore blood-based biomarkers that reliably reflect the pathological changes in the brain, avoiding the complexity of CSF sampling, as well as the high costs, radiation hazard and poor availability of PET neuroimaging [8]. A decreased blood plasma Aβ42/40 ratio has shown correlation to abnormal CSF Aβ concentrations [9] or positive amyloid-PET [10]; however, the magnitude of the difference in plasma is smaller than in the CSF [11]. It has been estimated that the Aβ42/40 ratio in CSF decreased by 50% in the presence of brain amyloidosis [12], whilst in plasma, it decreased by approximately 10–15% [10, 13]. Our recent work supports the hypothesis that selective measurement of plasma Aβ1-42 and Aβ1-40 (i.e. Aβ variants peptides starting with Asp(1)) and calculation of the Aβ1-42/1-40 ratio may allow for a diagnostic contrast enhancement [14].

Several assays for plasma Aβ42/40 have been reported to be able to identify individuals with abnormal brain Aβ burden, such as ultrasensitive immunoassays, fully automated immunoassays and immunoprecipitation mass spectrometry (IP-MS) approaches [10, 13, 15–18]. However, technical and analytical performance of the different platforms appears to differ to some extent [19]. Additionally, pre-analytical sample handling and cohort characteristics greatly contribute to variability between Aβ assays across studies [20].

In 2018, our group developed a “two-step immunoassay” approach consisting of magnetic bead immunoprecipitation (IP) of Aβ from EDTA–plasma followed by quantification of Aβ42 and Aβ40 by a commercially multiplex immunoassay kit (Mesoscale Discovery) to identify patients with AD [17]. In analogy to assay platforms combining IP-MS, this method can be referred to as IP with subsequent read-out by immunoassays (IP-IA). In an earlier study, it was shown that direct measurements of plasma Aβ42 and Aβ40 using the same MSD immunoassay performed poorly and did not reveal a statistically significant difference in the plasma Aβ42/40 ratio between amyloid-PET positive and negative individuals [21]. The effect of pre-analytical Aβ immunoprecipitation from plasma was further investigated in a pre-selected cohort analysed with fully automated prototype Elecsys® Aβ immunoassays (Roche Diagnostics GmbH). We observed that pre-analytical sample workup by Aβ-IP led to a statistically significant increase in the area under the receiver operating characteristics (ROC) curve (AUC) for the discrimination of subjects with low CSF Aβ42/40 from those with normal CSF Aβ42/40 [22]. In the present study, we investigated whether Aβ-IP from blood plasma was also compatible with fully automated Lumipulse G System (Fujirebio) plasma Aβ assays and if it might also improve the diagnostic performance.

In addition to the plasma Aβ42/40 ratio, several phosphorylated forms of Tau protein in blood plasma, namely pTau181, pTau231 and pTau217, were discovered to represent highly attractive biomarkers for AD [23–25]. To complement our studies on plasma IP as a pre-analytical sample workup aiming for attenuation of possible matrix interferences, we finally investigated if the IP-IA assay was also applicable to the analysis of plasma pTau181 on the Lumipulse platform. It is of note that very recently direct plasma biomarker measurements on the Lumipulse platform, including Aβ42/40 and pTau181, were shown to perform well in detecting AD pathological changes in CSF in cognitively unimpaired subjects [26].

## Materials and methods

### Study cohort and study approval

Study participants were recruited at the Department of Psychiatry and Psychotherapy at the University Medical Center Goettingen, from August 2016 to March 2020. Individual informed consent was required prior to study inclusion from all subjects or their legal representatives. Respective pseudonymized collection of biological samples and clinical data were conducted according to the revised Declaration of Helsinki and good clinical practice guidelines and their use in biomarker studies was approved by the ethics committee of the University Goettingen (9/2/16).

### Classification of the study participants

The pre-selected study cohort comprised 71 subjects representing a sub-set of a sample of originally *n = *80 subjects used in the previous studies [14, 22] and for whom sufficient EDTA–blood plasma was available in our local biobank. The study participants were diagnosed by a biomarker-supported clinical diagnosis [22] and dichotomized according to their CSF Aβ42/40 ratios measured with commercial Aβ-ELISA kits in the Laboratory of Clinical Neurochemistry and Neurochemical Dementia Diagnostics, Department of Psychiatry and Psychotherapy, University of Erlangen-Nuremberg, Germany. The subjects were classified according to the clinical cut-off point of 0.050 into the groups Aβ-positive (CSF Aβ42/40 ≤ 0.050, *n = *32) and Aβ-negative (CSF Aβ42/40 > 0.050, *n = *39). This classification was consistent with the biomarker-supported clinical diagnosis based on clinical evaluations [27], CSF biomarkers (Aβ ratio, pTau181 and total-Tau) and psychometric and neuroimaging biomarker data (16 of 71 patients), classifying all Aβ-positive participants as probable or possible AD and all other patients as Aβ-negative disease controls. In the group of Aβ-positive participants with possible or probable AD, 8 patients had mild cognitive impairment (MCI) and 24 patients had dementia, whilst in the group of Aβ negative participants with improbable AD, 1 had subjective cognitive decline (SCD), 23 had MCI and 15 had dementia. In this retrospective analysis of the participants, the evaluation of cognitive impairment was based on a combined neuropsychological and clinical examination. APOE status was determined using a modified quantitative real-time PCR protocol as described previously [28]. DNA was prepared from whole blood with the DNeasy Blood & Tissue kit (Qiagen) and analyses were performed on a CFX Connect Real-Time PCR system using the iTaq™ Universal SYBR Green Supermix (BIO-RAD). All samples were measured in duplicates for all primer combinations including negative controls. The characteristics of the study cohort and CSF biomarkers are summarised in Table 1.

**Table 1:** Description of the study cohort and baseline CSF biomarker data

	All (*n = *71)	Aβ-^a^ (*n = *39)	Aβ + ^a^ (*n = *32)	*p* value^b^
Age [years]	67 ± 8.9	66 ± 7.4	71 ± 6.7	0.031
Female	40 (56.3%)	21 (53.8%)	19 (59.4%)	0.810^c^
ApoE ε4 carrier	32 (45.1%)	7 (17.9%)	25 (78.1%)	< 0.001^c^
CSF Aβ42/40	0.069 ± 0.036	0.078 ± 0.009	0.036 ± 0.008	< 0.001
CSF Aβ42 [pg/mL]	742.0 ± 333.6	900.0 ± 271.3	475.5 ± 178.7	< 0.001
CSF Aβ40 [pg/mL]	12,051.0 ± 4106.8	11,271.0 ± 3266.2	12,531.0 ± 4808.8	0.132
CSF t-Tau [pg/mL]	316.0 ± 195.7	202.0 ± 71.2	476.5 ± 186.1	< 0.001
CSF pTau181 [pg/mL]^d^	49.6 ± 21.1	36.9 ± 10.8	74.55 ± 26.8	< 0.001

Numerical variables are reported as median ± median absolute deviation (MAD), categorical variables are reported as absolute and relative frequencies

*Aβ* amyloid-β, *CSF* cerebrospinal fluid

^a^The clinical sample was dichotomized according to the CSF Aβ42/40 ratio. Aβ-positive (Aβ +): CSF Aβ42/40 ≤ 0.050; Aβ-negative (Aβ −): CSF Aβ42/40 > 0.050

^b^Two-tailed Mann–Whitney test *p* values for the comparison between the groups Aβ + and Aβ −

^c^Two-tailed Fisher test *p* values for the comparison between the groups Aβ + and Aβ −

^d^For one subject, the CSF pTau181 concentration was < 15.6 pg/mL. For the statistical analysis, this value was artificially set to a fixed value of 15.6 pg/mL

### Preparation of functionalized magnetic beads

Functionalized magnetic beads for Aβ-immunoprecipitation (Aβ-IP) were produced by covalently coupling the monoclonal antibody (mAb) 1E8 (nanoTools, Teningen, Germany) with Dynabeads M-280 Sheep anti-Mouse IgG (Invitrogen/Thermo Fisher Scientific, Waltham, MA, USA) according to the manufacturer’s instructions and as described in detail previously [17].

For immunoprecipitation of Tau proteins, functionalized magnetic beads were produced by coupling two mAbs directed to Tau. First, mAb 2B8 was used (Roboscreen, Leipzig, Germany) that binds to brain derived Tau because its binding site is formed by the last 4 amino acids of exon 4 and the first 4 amino acids of exon 5. This epitope contains amino acids 121–128 (HVTQARMV) of 2N4R Tau. Second, mAb 7E5 (Roboscreen) was used which is directed to amino acids RGAAPPGQKGQA (156–165 of 2N4R Tau). Dynabeads M-270 Epoxy (Invitrogen/ Thermo Fisher Scientific) were coupled with both mAbs according to the manufacturer’s protocol using 100 µg of mAb per 5 mg (~ 3.3 × 10^8^) of lyophilized beads. Coupling was performed at 37 °C using rotation at 25 rounds per minute overnight (12–18 h) in a mixture of 20 mM phosphate-buffered saline pH 7.4 (PBS), 0.1 M sodium phosphate buffer pH7.4 and 3 M ammonium sulphate in 0.1 M sodium phosphate buffer pH7.4 (v/v 1/1/1). After coupling, the beads were washed 3 times using 20 mM PBS pH 7,4 containing 0,1% bovine serum albumin (BSA) und 0,05% (v/v) Tween 20 and stored in the same buffer supplemented with 0.02% sodium azide or 0.1% proclin300.

### Semi‑automated Aβ and Tau‑immunoprecipitations

Plasma Aβ peptides were immunoprecipitated from EDTA–blood plasma in a semi-automated fashion on a CyBio FeliX liquid handling instrument (Analytik Jena, Jena, Germany) following a modified version of our previously published Aβ IP protocol [14]. In brief, aliquots of 500 µl EDTA–blood plasma stored at –80 °C in Matrix 0.5 mL tubes (Thermo Scientific) were thawed at room temperature (RT), mixed vigorously for 5–10 s and centrifuged for 10 min at 10,000 × *g* at RT in a fixed angle rotor for removal of insoluble material. Next, 200 µL of plasma was transferred manually to a 96-deepwell sample plate (DeepWell MegaBlock®, 96 wells, 2.2 mL, PP (Sarstedt, Nümbrecht, Germany)) and placed inside the CyBio FeliX instrument. The plasma samples were mixed with 200 µL H_2_O, 100 µL of 5 × IP buffer concentrate (250 mM HEPES/NaOH, pH 7.4, 750 mM NaCl, 2.5% Igepal CA630, 1.25% sodium deoxycholate; 0.25% SDS and Complete Mini Protease inhibitor cocktail (Roche)) and 25 µL of functionalized 1E8 magnetic beads (see above), followed by overnight incubation at 4 °C with continuous agitation at 1,000 rpm on an Eppendorf ThermoMixer C (Eppendorf, Hamburg, Germany). On the next day, the incubated plate was placed in the FeliX instrument for subsequent washing steps. The magnetic beads were then immobilised with an ALPAQUA MAGNUM FLX Universal Magnet (Beverly, MA, USA) adapter and the supernatants (unbound material) were discarded. The beads were immediately washed 3 × for 5 min with 1 mL of PBS/0.1% BSA and 1 × for 3 min with 1 mL of 10 mM Tris/HCl, pH 7.5. Per well, 2 × 25 µL of PBS containing 0.05% Tween 20 (PBS-T) were used to elute the Aβ peptides from the 1E8 magnetic beads by heating the 96-deepwell round bottom plate without a lid for 5 min at 99 °C and 1,100 rpm on a BioShake 3000-T elm (QInstruments, Germany) mounted in the CyBio FeliX instrument. Per sample, a remaining volume of approximately 38 µL of bead-free Aβ eluate was obtained and diluted ~ six-fold with 190 µL of Diluent 35 (MSD). Finally, the diluted Aβ IP eluates were divided into three aliquots of 60µL and stored at − 80 °C until the Aβ measurements on the Lumipulse G System.

For the immunoprecipitation of Tau proteins, we started from 400 µL aliquots of EDTA–plasma. First, the plasma samples were depleted of Aβ peptides. For that purpose, 400 µL of EDTA–plasma was mixed with 100 µL of 5 × IP buffer concentrate and 25 µL 1E8 magnetic beads on the CyBio FeliX liquid handling robot (see above). After overnight incubation, the magnetic beads were immobilised and the unbound fractions (supernatants) were collected and stored at 4 °C for subsequent Tau IP. To each of the precleared samples, 50 µL of Tau beads (described above) were added, followed by an overnight incubation at RT with periodical mixing by pipetting up and down on the CyBio FeliX instrument. After the incubation, the beads were collected on the ALPAQUA MAGNUM FLX Universal Magnet, and the supernatants were discarded. The beads were washed 3 × for 5 min with 1 mL of PBS/0.1% BSA and 1 × for 3 min with 1 mL of PBS-T. Finally, Tau proteins were eluted in 2 × 25 µL of PBS-T by heating the 96-deepwell round bottom plate without a lid for 5 min at 99 °C and 1,100 rpm in a BioShake 3000-T elm (QInstruments, Germany). To the remaining volume of approximately 38 µL of Tau IP eluate per sample, 75 µl of PBS-T was added, resulting in a final volume of approximately 113 µl per sample. The eluates were divided into two aliquots of 50 µl and stored in 0.5 mL Protein LoBind tubes (Eppendorf) at − 80 °C until pTau181 measurements on the Lumipulse G System.

### Quantification of Aβ1-40, Aβ1-42 and pTau181 on Lumipulse

The concentrations of Aβ1-40, Aβ1–42 and pTau181 were determined using the commercially available plasma β-Amyloid 1-40, 1–42 and pTau181 Immunoreaction Cartridges on the fully automated Lumipulse G600II System. A new calibration curve was performed for each analyte before the experiment. Quality control analyses were performed every start of the day using Aβ and pTau181 control samples included in the kit.

For the direct measurements of EDTA–plasma, 400 µL of centrifuged plasma was introduced into the instrument using individual 2 mL screw cap micro tubes (Sarstedt, Germany). Single measurements of Aβ1-40, Aβ1–42 and pTau181 were performed consecutively by the automated Lumipulse system using the same sample.

IP eluate analysis was divided in two steps. Quantification of Aβ1-40 and Aβ1–42 was performed by diluting one of the three 60 µL aliquots of Aβ IP eluates stored at -80 °C to a final volume of 300 µL using Specimen Diluent 1 (Fujirebio). Tau IP eluate was used to measure the levels of pTau181. A sample of 50 µL, corresponding to approximately half of the eluate volume, was diluted to a final volume of 250 µL. Diluted samples were transferred to 2 mL Sarstedt tubes and introduced into the Lumipulse instrument for the respective measurement. All assays were performed as single measurements following the kit instructions.

### Statistical analysis

All statistical evaluations were performed with R version 4.2.3. Baseline statistics are reported as median ± median absolute deviations with scaling factor 1.4826 (MAD). To compare between amyloid-positive and amyloid-negative groups, we used two-tailed Mann–Whitney tests for numerical variables and Fisher’s exact test for categorical variables. For the calculation of correlation coefficients, we used Pearson correlations. For fitting regression lines, we used a Deming regression (R package MethComp version 1.22.2) since both variables were measured experimentally.

For assessing effect sizes, we used three parameters as measures of the magnitude of the effect:

(i) The relative median difference, calculated as:

$$\mathrm{Median difference }\left(\mathrm{\%}\right)=100\times \frac{\mathrm{median }(\mathrm{A\beta }+)-{\text{median}}(\mathrm{A\beta }-)}{{\text{median}}(\mathrm{A\beta }-)}$$

(ii) The relative mean difference, calculated as:

$$\mathrm{Mean difference }\left(\mathrm{\%}\right)=100\times \frac{\mathrm{mean }\left(\mathrm{A\beta }+\right)-{\text{mean}}(\mathrm{A\beta }-)}{\mathrm{mean }(\mathrm{A\beta }-)}$$

(iii) Cohen’s d (standardised effect size), calculated with R package “effsize” (version 0.8.1).

For testing the significance of the observed difference of effect sizes, we applied a 0.632 bootstrapping (re-sampling of patients with replacement including refinement of the estimator as proposed by Efron in 1983) [29]. We applied 1000 replications of the bootstrapping and calculated the difference of effect sizes (e.g. 0.632 × median difference of the resampling + 0.368 × median difference of data without resampling). The differences of the resulting effect sizes were normally distributed (Shapiro p value: 0.85). Making use of this fitted normal distribution, bootstrapping p values were calculated using a normal distribution after normalisation of the standard deviation. Single value ROC curves, AUCs and Delong p value for comparing ROC curves were calculated with R package pROC (version 1.18.0). Classification with logistic regression was done with a tenfold cross-validation in order to avoid overfitting. Classification performance was assessed at the Youden point of the ROC curve. Mixture models were used to fit two Gaussian distributions for the Aβ1-42/1-40 ratios. R package ‘mixtools’ (version 2.0.0) was used with the precondition of similar standard deviations between the two groups.

## Results

### Plasma Aβ measurements without and with pre-analytical Aβ IP

**Fig. 1:**
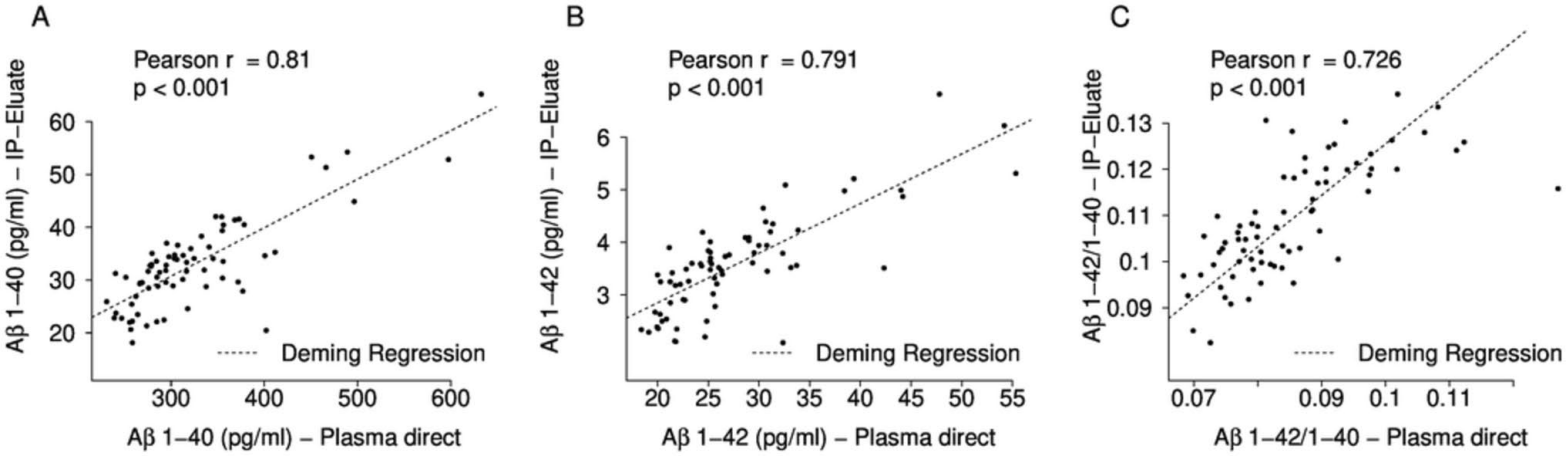
Correlations between untreated plasma and IP-Eluate of **A** Aβ1-40, **B** Aβ1-42 and **C** Aβ1-42/1-40 ratio measurements on Lumipulse. Diluted IP-Eluate concentrations are plotted against the corresponding direct plasma measurement. The diagonal dashed lines correspond to the Deming regressions. Pearson correlation coefficients and p values are indicated. *Aβ* amyloid-β, *IP* immunoprecipitation

EDTA–plasma samples from 71 individuals were analysed on the Lumipulse G600II platform by single measurements of undiluted plasma and in eluates obtained after Aβ-IP from 200 µL of plasma. The measured concentrations of Aβ1-40 and Aβ1-42 were above the published lower limits of quantification (LoQ) (Aβ1-40: 0.44 pg/mL and Aβ1-42: 0.43 pg/mL) [30] for all samples analysed. Considerably lower levels of Aβ1-40 and Aβ1-42 peptides were detected in the Aβ-IP eluates as compared to direct plasma measurements (Fig. 1A and B). This was expected and can be explained to some extent by the increase in volume caused by the IP procedure and the sample preparation for the immunoassay: starting from 200 µL of EDTA–plasma, approximately 38 µL of IP eluate was obtained. For storage (in aliquots), 190 µL of Diluent 35 was added. Prior to the measurements on Lumipulse, the samples were diluted fivefold (see above) resulting in an overall dilution by a factor of approximately 5.7. The actual measured concentrations (means) of Aβ1-40 and Aβ1-42 were 9.8-fold and 7.7-fold higher respectively in direct plasma measurements than after IP, indicating incomplete Aβ recoveries after IP. As explained previously, the pre-analytical Aβ-IP protocol aimed for an amelioration of potential matrix effects, but not for concentration of the Aβ peptides prior to the measurements [22]. The concentrations of both Aβ isoforms measured in plasma without pre-treatment were positively and strongly correlated to the corresponding concentrations found in the diluted Aβ-IP eluates (Pearson’s *ρ = *0.810, *p < *0.001 for Aβ1-40, Fig. 1A; Pearson’s *ρ = *0.791, *p < *0.001 for Aβ1-42, Fig. 1B). The calculated Aβ1-42/1-40 ratios showed a positive and strong correlation between direct plasma measurements and the respective IP eluate measurements (Pearson’s *ρ = *0.726, *p < *0.001, Fig. 1C).

### Plasma pTau181 measurements without and with pre-analytical Tau IP

**Fig. 2:**
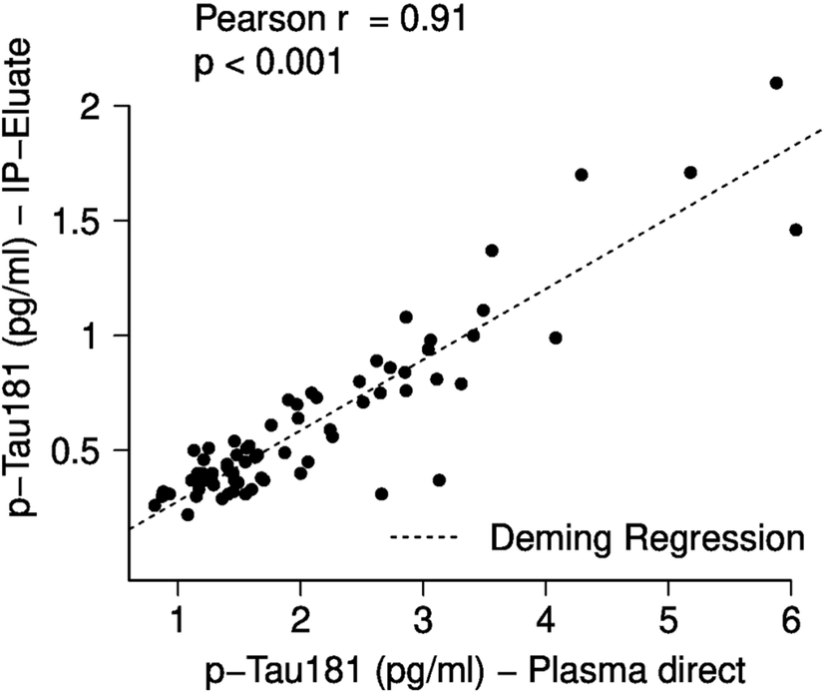
Correlations between untreated plasma and IP-Eluate of pTau181 measurements on Lumipulse. Diluted IP-Eluate concentrations are plotted against the corresponding direct plasma measurement. The diagonal dashed lines correspond to the Deming regression. Pearson correlation coefficients and p-values are indicated. IP immunoprecipitation

Next, plasma pTau181 levels were analysed in the same cohort (*n = *71) by direct plasma measurements and after pre-analytical sample workup consisting of Aβ depletion followed by Tau-IP with a combination of phosphorylation-insensitive pan Tau antibodies. Overall, the Tau-IP procedure and sample preparation led to a ~ 1.4-fold sample dilution compared to the original plasma volume. Assuming 100% recovery after Tau-IP, the expected concentrations of pTau181 in the IP eluates would be 1/1.4 = 71% of those in the direct plasma measurements. However, the mean pTau181 concentration found in the IP eluates accounted for only 29% of the direct measurements, indicating incomplete recoveries. Nevertheless, a strong correlation was observed between the concentrations of pTau181 measured in plasma without pre-treatment and those in the Tau IP eluates (Pearson’s *ρ = *0.910, *p < *0.001, Fig. 2). All measurements were above the published LoQ of the assay (0.134 pg/mL) [31].

### Aβ and pTau181 plasma measures in diagnostic groups

**Fig. 3:**
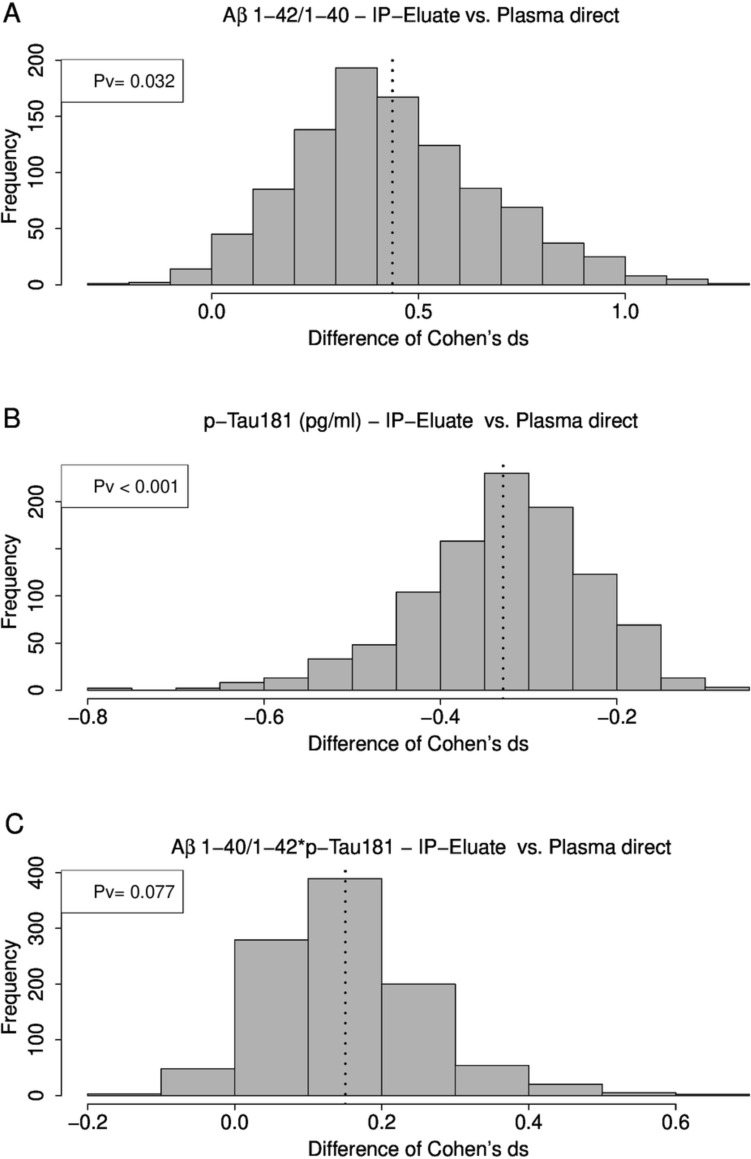
Bootstrapping experiment for the comparison of effect sizes (Cohen’s d) for plasma (**A**) Aβ1-42/1-40, (**B**) pTau181 and the (**C**) Aβ1-40/1–42*pTau181 ratio with and without pre-analytical sample workup by IP. *Aβ* amyloid-β, *IP* immunoprecipitation

**Fig. 4:**
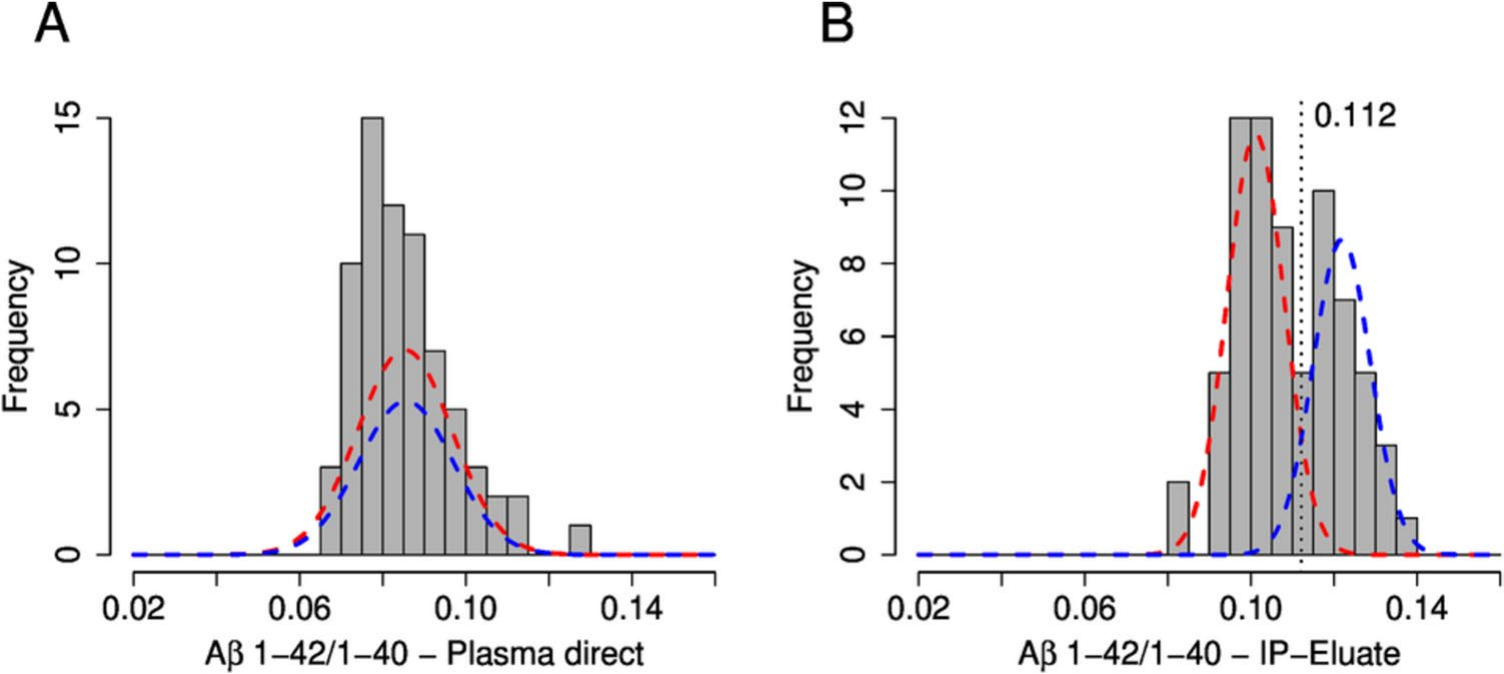
Unsupervised mixed model histograms of the distribution of the Aβ1-42/1-40 ratios measured in (**A**) EDTA–plasma and (B) plasma Aβ-IP eluates. Two Gaussian distributions were fitted to the distribution using an unsupervised mixture model approach. The vertical dashed line in (**B**) shows the intersection of the two curves at a Aβ1-42/1-40 ratio of 0.112 (threshold). *Aβ* amyloid-β, *IP* immunoprecipitation

The preselected participants in this study were classified according to their CSF Aβ42/40 ratios into the diagnostic groups Aβ-positive (CSF Aβ42/40 ≤ 0.050, *n = *32) and Aβ-negative (CSF Aβ42/40 > 0.050, *n = *39). This classification was consistent with the biomarker-supported clinical diagnosis: all Aβ-positive subjects were diagnosed as possible/probable AD whilst all Aβ-negative subjects were classified as disease controls. There was no statistically significant group difference in plasma Aβ1-40 when measured either directly or after pre-analytical Aβ-IP (Table 2). The measured plasma concentrations of Aβ1-42 and the Aβ1-42/1-40 ratio were statistically significantly lower in the Aβ-positive group. For the Aβ1-42/1-40 ratio, the magnitude of the difference in medians between the diagnostic groups was -14.0% (Aβ-positive vs. Aβ-negative) in direct plasma measurements and − 15.9% in Aβ-IP eluates. The mean difference between the study groups was − 15.7% in direct plasma compared to − 15.0% in IP eluate measurements. The calculated standardised effect sizes (Cohen’s d values) for the plasma Aβ1-42/1-40 ratio were 1.62 for direct plasma measurements and 2.03 for Aβ-IP eluates. Application of a 0.632 bootstrapping experiment (re-sampling from the study participants with 1000 replications) showed that the Aβ-IP led to a significant increase in the standardised effect size (Cohen’s d) with a p value of 0.0331 (Fig. 3A). Using an unsupervised mixture model approach, a bimodal distribution in the Aβ1-42/1-40 ratios was observed in the measurements after Aβ-IP, but not in direct plasma. For the former, two Gauss curves were fitted showing an intersection at an Aβ1-42/1-40 ratio of 0.112 (Fig. 4).

**Table 2:** Plasma measures of Aβ isoforms and pTau181 and calculated Aβ1-42/1-40 ratio and Aβ1-40/1–42*pTau181 ratio with and without pre-analytical IP in amyloid-positive and amyloid-negative subjects

	Variable	Median Aβ-	Mean Aβ-	MAD^a^ Aβ-	Median Aβ +	Mean Aβ +	MAD^a^ Aβ +	P-value^b^	Median Diff (%)	Mean Diff (%)	Cohens’ d
Direct plasma	Aβ1-40	303.7	329.5	61.6	293.8	316.4	44.9	0.337	− 3.3	− 4.0	0.169
	Aβ1-42	29.0	30.2	5.3	22.6	24.6	3.8	< 0.001	− 22.1	− 18.4	0.754
	Aβ1-42/1-40	0.090	0.092	0.009	0.077	0.076	0.005	< 0.001	− 14.0	− 15.7	1.621
	pTau181	1.45	1.58	0.37	2.55	2.71	0.91	< 0.001	75.9	71.8	-1.170
	$\frac{{\text{A}}\upbeta 1-40}{{\text{A}}\upbeta 1-42}*{\text{pTau}}181$	15.32	17.32	5.042	32.34	35.10	12.525	< 0.001	111.1	102.7	-1.472
IP-Eluate	Aβ1-40	31.9	34.0	4.0	32.2	31.6	6.9	0.430	0.8	-6.9	0.270
	Aβ1-42	3.80	3.94	0.59	3.10	3.13	0.82	< 0.001	− 18.4	− 20.5	0.957
	Aβ1-42/1-40	0.118	0.117	0.010	0.100	0.100	0.005	< 0.001	− 16.0	− 15.0	2.027
	pTau181	0.380	0.415	0.103	0.755	0.859	0.304	< 0.001	98.7	107.0	-1.480
	$\frac{{\text{A}}\upbeta 1-40}{{\text{A}}\upbeta 1-42}*{\text{pTau}}181$	3.45	3.56	0.701	7.44	8.76	3.198	< 0.001	115.4	145.9	-1.600

*Aβ* amyloid-β, *IP* immunoprecipitation

^a^MAD: median absolute deviation scaled with a factor 1.4826

^b^Mann–Whitney test p values for the comparison between the groups Aβ-positive (Aβ +) and Aβ-negative (Aβ −)

The pTau181 levels measured on Lumipulse with or without pre-analytical Tau-IP were statistically significantly increased in the Aβ-positive group compared to the Aβ-negative group (Table 2). In direct plasma measurements, a difference between both diagnostic groups of 75.9% in the medians and 71.8% in the means was observed. In the Tau-IP eluates, the magnitude of the difference in medians between the diagnostic groups was 98.7% and 107% for the difference in means. The Cohen’s d values were (−)1.17 for direct measurements and (−)1.48 after pre-analytical Tau-IP. Resampling in a 0.632 bootstrapping experiment revealed a p-value of *p < *0.001 (Fig. 3B) for the observed improvement in Cohen’s d after Tau-IP.

### Single value receiver operating characteristics analysis

To compare the classification performance of the plasma biomarker measurements on Lumipulse with and without pre-analytical IP, we calculated single value ROC curves for Aβ1-42/1-40 and pTau181. Additionally, we tentatively assessed $\frac{\mathrm{A\beta }1-40}{\mathrm{A\beta }1-42}*{\text{pTau}}181$ as a novel term (AT-term) combining Aβ and pTau biomarkers. The results of the ROC analysis and the classification statistics for the differentiation between Aβ-positive and Aβ-negative subjects are summarised in Table 3.

**Table 3:** Statistics for single value ROC analyses evaluated at the Youden point

Variable	AUC (95%CI)	Tp	Tn	Fp	Fn	Ppv	Npv	Sensitivity	Specificity	Accuracy
Aβ1-42/1-40 (plasma direct)	0.907 (0.834–0.980)	30	32	7	2	0.811	0.941	0.938	0.821	0.873
Aβ1-42/1-40 (Aβ-IP eluate)	0.934 (0.868–1.000)	32	32	7	0	0.821	1.000	1.000	0.821	0.901
pTau181 (plasma direct)	0.817 (0.717–0.916)	25	31	8	7	0.758	0.816	0.781	0.795	0.789
pTau181 (Tau-IP eluate)	0.894 (0.809–0.979)	28	35	4	4	0.875	0.897	0.875	0.897	0.887
$\frac{{\text{A}}\upbeta 1-40}{{\text{A}}\upbeta 1-42}*{\text{pTau}}181$(plasma direct)	0.887 (0.814–0.960)	29	28	11	3	0.725	0.903	0.906	0.718	0.803
$\frac{{\text{A}}\upbeta 1-40}{{\text{A}}\upbeta 1-42}*{\text{pTau}}181$(Tau-IP eluate)	0.929 (0.864–0.993)	30	35	4	2	0.882	0.946	0.938	0.897	0.915

*Aβ* amyloid-β, *AUC* %CI confidence interval, *Tp* true positive, *Tn* true negative, *Fp* false positive, *Fn* false negative, *Ppv* positive predictive value, *Npv* negative predictive value, *IP* immunoprecipitation

**Fig. 5:**
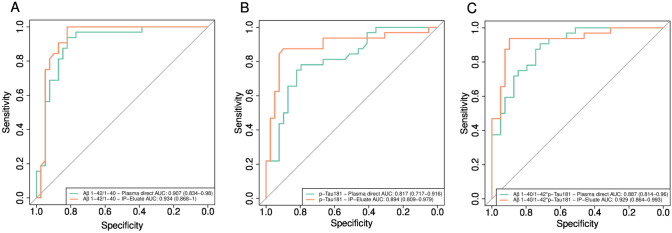
Pairwise comparisons of single value ROC curves for plasma biomarkers measured directly or after pre-analytical IPs on the Lumipulse platform. ROC curves for the discrimination between amyloid-positive and amyloid-negative study participants were calculated for **A** Aβ1-42/1-40, **B** pTau181 and **C** $\frac{\mathrm{A\beta }1-40}{\mathrm{A\beta }1-42}*{\text{pTau}}181$. Aβ amyloid-β, IP immunoprecipitation

Pre-analytical Aβ-IP or/and Tau-IP increased the numerical values of the areas under the ROC curves (AUCs) for all of the three tested biomarkers. However, only in the case of plasma pTau181, a *p* value < 0.05 was observed after performing pairwise DeLong tests (*p = *0.038) without correction for multiple comparisons (Fig. 5). Noteworthy, for the AT-term, the diagnostic accuracy increased from 0.803 for direct measurements to 0.915 for IP-IA, which was mainly due to an improvement in diagnostic specificity.

### Multivariate logistic regression

To assess whether the detection of amyloid positivity based on the plasma Aβ1-42/1-40 ratio measured after pre-analytical Aβ IP could be further improved by considering ApoE4 status and plasma pTau181 (measured in Tau-IP eluates), we performed multivariate ROC analyses by logistic regression with 10 leave-out cross-validation. The numerical logistic regression ROC-AUC for Aβ1-42/1-40 was 0.927 (95% CI = 0.859–0.996), confirming the AUC from single-value ROC analysis (see above). Including plasma pTau181 (measured after Tau-IP) or ApoE4 status in the analysis increased the AUC to 0.946 (95% CI = 0.889–1.0) or 0.950 (95% CI = 0.894–1.0), respectively. Considering all three parameters yielded an AUC of 0.952 (95% CI = 0.897–1.0). None of the numerical increases in the AUC values reached statistical significance according to pairwise comparisons with DeLong tests. The statistics of the logistic regression analysis with 10 leave-out cross-validation are shown in Table 4.

**Table 4:** Statistics of ROC analysis by logistic regression with 10 leave-out cross-validation

Variable	AUC (95%CI)	Tp	Tn	Fp	Fn	Ppv	Npv	Sensitivity	Specificity	Accuracy
Aβ1-42/1-40	0.927 (0.859–0.996)	32	32	7	0	0.821	1.000	1.000	0.821	0.901
Aβ1-42/1-40 + pTau181	0.946 (0.889–1.000)	31	34	5	1	0.861	0.971	0.969	0.872	0.946
Aβ1-42/1-40 + ApoE4 status	0.950 (0.894–1.000)	31	35	4	1	0.886	0.972	0.969	0.897	0.930
Aβ1-42/1-40 + pTau181 + ApoE4 status	0.952 (0.897–1.000)	32	35	4	0	0.889	1.000	1.000	0.897	0.944
$\frac{{\text{A}}\upbeta 1-40}{{\text{A}}\upbeta 1-42}*{\text{pTau}}181$	0.909 (0.833–0.986)	28	36	3	4	0.903	0.900	0.875	0.923	0.901
$\frac{{\text{A}}\upbeta 1-40}{{\text{A}}\upbeta 1-42}*{\text{pTau}}181$+ ApoE4 status	0.953 (0.907–0.999)	32	31	8	0	0.800	1.000	1.000	0.795	0.887

Logistic regression analysis was done for the Lumipulse measurements of plasma Aβ1-42/1-40 in Aβ-IP eluates and plasma pTau181 in Tau-IP eluates. ApoE4 status was determined by QPCR. The classification statistics were done at the maximum Youden index

*Aβ* amyloid-β, *AUC* %CI confidence interval, *Tp* true positive, *Tn* true negative, *Fp* false positive, *Fn* false negative, *Ppv* positive predictive value, *Npv* negative predictive value, *IP* immunoprecipitation

### Correlations between plasma and CSF: Aβ and pTau181

**Fig. 6:**
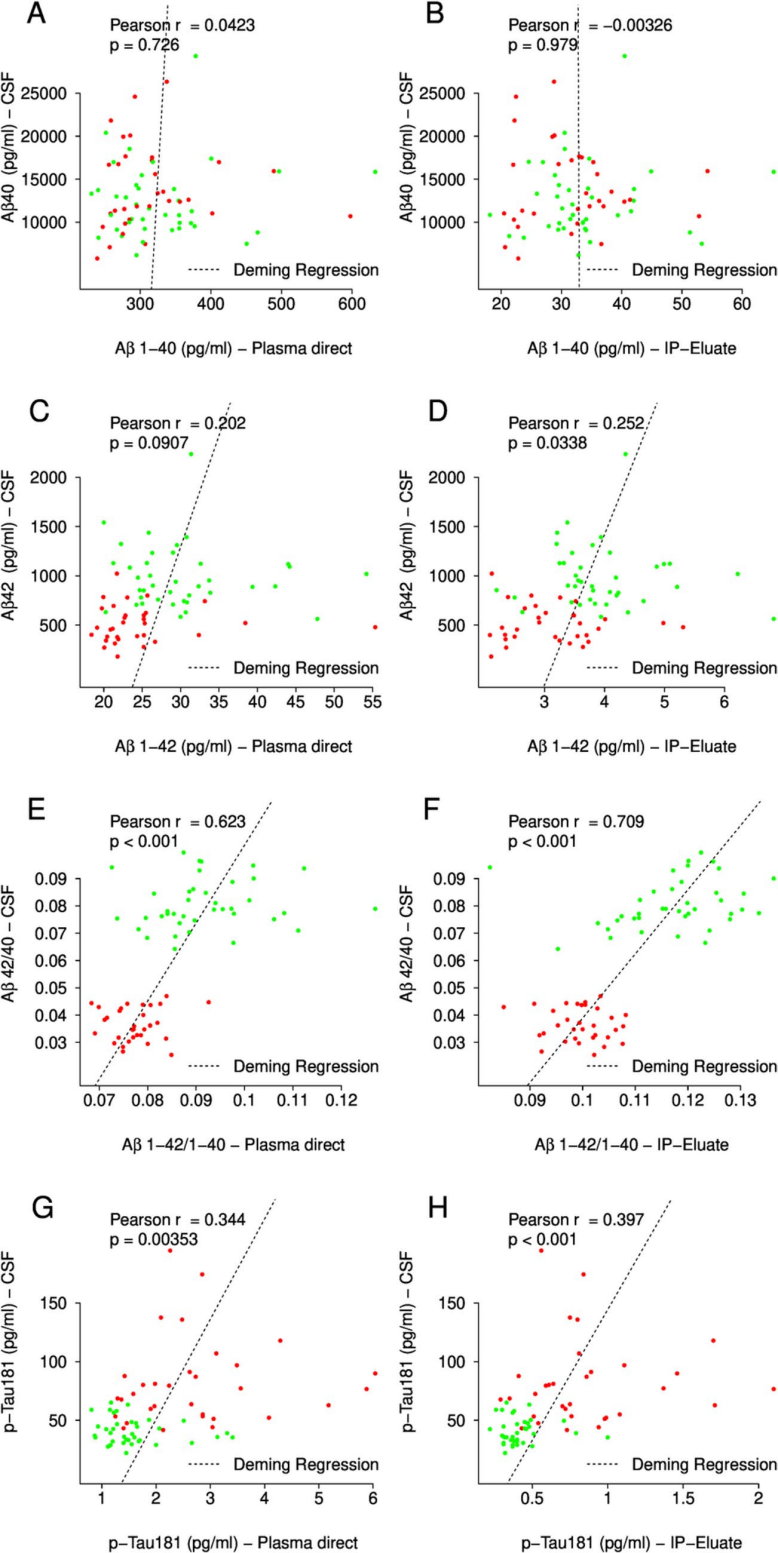
Correlations between CSF ELISA data and the corresponding plasma values measured with Lumipulse directly or after pre-analytical magnetic bead IP. CSF concentrations of **A**, **B** Aβ1-40, **C**, **D** Aβ1-42, **E**, **F** Aβ1-42/1-40 ratio and **G**, **H** pTau181 are plotted against the corresponding plasma values measured on untreated plasma or IP eluate, respectively. Red = Aβ + and gree*n = *Aβ − . The diagonal dashed lines correspond to the Deming regressions. Pearson correlation coefficients and *p* values are indicated. Aβ amyloid-β, IP immunoprecipitation, CSF cerebrospinal fluid

Aβ1-40, Aβ1-42 and pTau181 measured directly in plasma and IP eluates and the calculated Aβ1-42/1-40 ratios were compared to the corresponding CSF levels (Fig. 6). There was no statistically significant correlation between CSF Aβ40 and plasma Aβ1-40 in direct measurements or in Aβ-IP eluates (Fig. 6A and B). CSF Aβ42 was statistically significantly correlated with Aβ1-42 in IP eluates but not in direct plasma measurements (Fig. 6C and D). The CSF Aβ42/40 ratio was statistically significantly correlated with plasma Aβ1-42/1-40 measured directly (*ρ = *0.623, *p < *0.001) and after Aβ-IP (*ρ = *0.709, *p < *0.001) (Fig. 6E and F). The correlations between CSF pTau181 and plasma pTau181 in direct measurements (*ρ = *0.344, *p = *0.004) and after Aβ depletion and Tau-IP (*ρ = *0.397, *p < *0.001), were significantly lower as compared to the Aβ peptide ratios (Fig. 6G and H).

## Discussion

Pre-analytical immunoprecipitation of Aβ peptides was previously shown to improve the diagnostic performance of automated prototype Elecsys plasma Aβ assays for detecting low CSF Aβ42/40 [22]. In the current study, we have investigated if automated Aβ and pTau181 measurements on the Lumipulse G System were also compatible with semi-automated pre-analytical Aβ and Tau-IP and to what extent this additional sample workup might impact the diagnostic performance of the plasma biomarkers Aβ1-42/1-40 and pTau181.

In direct plasma Aβ measurements on Lumipulse and after pre-analytical Aβ-IP (“IP-IA”), the median and mean Aβ1-42/1-40 ratios in the Aβ-positive group were decreased to a comparable extent in the range of 14–16%. The standardised effect size (Cohen`s d) increased statistically significantly after pre-analytical Aβ IP from 1.621 (direct plasma measurements) to 2.027 (IP-IA) (*p = *0.03, 0.632 bootstrapping). It appears that pre-analytical plasma treatment reduced the intra-group variances in terms of Aβ1-42/1-40, thereby increasing the standardised effect size. ROC analysis for the discrimination between Aβ-positive and Aβ-negative subjects showed a numerical increase in the AUC after Aβ-IP from 0.907 to 0.934 that did not reach statistical significance (*p = *0.35).

The performance of pTau181 measured on Lumipulse appeared to profit substantially and in a statistically significant degree from pre-analytical Tau-IP. In direct plasma measurements, the median and mean pTau181 levels in Aβ-positive subjects were increased by 76 and72%, respectively, compared to the Aβ-negative group. An accentuated increase by 98% in the median and 107% in mean levels of pTau181 was observed in the plasma Tau-IP eluates. Moreover, the standardised effect size (Cohen’s d) was (-)1.48 in IP eluates as compared to (-)1.17 in direct plasma measurements (*p < *0.001, 0.623 bootstrapping). The ROC-AUC increased significantly after pre-analytical Tau-IP from 0.817 to 0.894 (*p = *0.039).

As a novel term combining the plasma measurements of Aβ1-40, Aβ1-42 and pTau181, we propose the ratio $\frac{A\upbeta 1-40}{A\upbeta 1-42}*{\text{pTau}}181$ (“AT-term”) which may provide a single cut-point for the combination of both amyloid and Tau biomarkers. Regarding a possible future clinical application it is noteworthy that the diagnostic accuracy of the AT-term in our sample increased from 0.803 (direct plasma measurements) to 0.912 after pre-analytical Aβ and Tau-IPs (IP-IA). However, neither the standardised effect size (Cohen´s d) nor the ROC-AUC for the AT-term was statistically significantly different between direct measurements and IP-IA. Further studies in larger and independent cohorts will be required to investigate whether the AT-ratio may provide an added diagnostic value. In view of a number of recent studies indicating that pTau217 is a particularly promising AD biomarker, future work should furthermore address the question whether substituting plasma pTau181 by pTau217 in IP-IA may further improve the diagnostic value of the AT-ratio.

Overall, in the small and carefully pre-selected sample that was studied here, we observed that the IP-IA protocol on Lumipulse improved the accuracy to predict CSF biomarker evidence of amyloid deposition compared to direct plasma measurements. ROC analysis by multivariate logistic regression showed a maximum AUC of 0.952 for the discrimination between the groups in this selected cohort by combining the three analytes Aβ1-42/1-40 ratio, pTau181 and ApoE4 status. However, it was not statistically significantly better than the model with Aβ1-42/1-40 ratio alone (AUC = 0.927, *p = *0.256) or Aβ1-42/1-40 + pTau181 (AUC = 0.946, *p = *0.705).

Notwithstanding the good correlation between direct plasma measurements and IP-IA, the measured concentrations of Aβ1-40, Aβ1-42 and pTau 181 in IP eluates were substantially lower in IP eluates than in untreated plasma. The lower levels of Aβ peptides and pTau181 can be explained by incomplete recoveries after IP and an increase in the volume resulting from the particular assay protocols that were used. Importantly, the pre-analytical sample workup was not intended to pre-concentrate the analytes but to ameliorate potential matrix effects [22]. Taken together, our observations indicate that depletion of plasma proteins, such as albumin, IgG or other potential interfering substances, by immunoprecipitation resulted in a better diagnostic contrast. To the best of our knowledge, this was the first time plasma pTau181 was investigated by Tau immunoprecipitation followed by immunoassay measurement (IP-IA protocol). The novel pre-analytical treatment significantly improved the diagnostic performance of this plasma biomarker on the Lumipulse platform.

Interesting to mention, in an unsupervised mixed model approach, the IP-IA showed a bimodal distribution in plasma Aβ1-42/1-40, which was not observed for untreated direct plasma measurements. This allowed calculating two normal distributions showing an intersection at an Aβ1-42/1-40 ratio of 0.112 in this pilot cohort. Determination of diagnostic threshold values (cutpoints) for CSF Aβ1-42 by Gaussian mixture modelling was introduced previously as an unbiased approach to address an observed gradual drift in CSF Aβ1-42 values measured with the Innotest ELISA over two decades [32]. The cross section obtained for the plasma IP-IA Aβ1-42/1-40 ratio by mixed modelling in our study (0.112) was very close to the corresponding cut-point of 0.109 determined by single value ROC curve analysis at the Youden point.

In summary, the observations from this study and previous work [22] suggest that pre-analytical sample workup by IP may improve the ability of immunological measurements of plasma AD biomarkers, such as Aβ42/40 ratio and pTau, to predict brain amyloid pathology. However, whilst the proposed IP-IA approach worked reasonably well in our hands in a small cohort, widespread use or implementation in clinical immunoassay application may be hampered by the time-consuming sample pre-treatment, additional costs, the risk of added technical variability and the need for a high level of standardisation and automatization. Further limitations of the current study include the small sample size and the use of a highly pre-selected study cohort.

In conclusion, pre-analytical immunoprecipitation of blood plasma samples appears to have the potential to improve the diagnostic performance of specific plasma AD biomarker immunoassays. Plasma Aβ1-42/1-40 ratio was the best single predictor of AD neuropathological changes in our sample. Yet, the combination with pTau181 and ApoE showed a slight improvement in predicting low CSF Aβ42/40 as a proxy of Aβ status in the brain.

## Data Availability

The datasets used and/or analysed in the present study are available from the corresponding authors on reasonable request.

## References

[1] Garre-Olmo J (2018) Epidemiology of Alzheimer’s disease and other dementias. Rev Neurol 66:377–386 29790571

[2] Masters CL, Simms G, Weinman NA, Multhaup G, McDonald BL, Beyreuther K (1985) Amyloid plaque core protein in Alzheimer disease and down syndrome. Proc Natl Acad Sci 82:4245–4249 3159021 10.1073/pnas.82.12.4245 PMC397973

[3] Grundke-Iqbal I, Iqbal K, Tung Y-C, Quinlan M, Wisniewski HM, Binder LI (1986) Abnormal phosphorylation of the microtubule-associated protein tau (tau) in Alzheimer cytoskeletal pathology. Proc Natl Acad Sci 83:4913–4917 3088567 10.1073/pnas.83.13.4913 PMC323854

[4] Van Dyck CH, Swanson CJ, Aisen P, Bateman RJ, Chen C, Gee M, Kanekiyo M, Li D, Reyderman L, Cohen S (2023) Lecanemab in early Alzheimer’s disease. N Engl J Med 388:9–21 36449413 10.1056/NEJMoa2212948

[5] Jack CR, Bennett DA, Blennow K, Carrillo MC, Feldman HH, Frisoni GB, Hampel H, Jagust WJ, Johnson KA, Knopman DS (2016) A/t/n: An unbiased descriptive classification scheme for Alzheimer disease biomarkers. Neurology 87:539–547 27371494 10.1212/WNL.0000000000002923 PMC4970664

[6] Dubois B, Feldman HH, Jacova C, Hampel H, Molinuevo JL, Blennow K, DeKosky ST, Gauthier S, Selkoe D, Bateman R (2014) Advancing research diagnostic criteria for Alzheimer’s disease: the iwg-2 criteria. Lancet Neurol 13:614–629 24849862 10.1016/S1474-4422(14)70090-0

[7] Ebenau JL, Timmers T, Wesselman LM, Verberk IM, Verfaillie SC, Slot RE, Van Harten AC, Teunissen CE, Barkhof F, Van Den Bosch KA (2020) Atn classification and clinical progression in subjective cognitive decline: the science project. Neurology 95:e46–e58 32522798 10.1212/WNL.0000000000009724 PMC7371376

[8] Brand AL, Lawler PE, Bollinger JG, Li Y, Schindler SE, Li M, Lopez S, Ovod V, Nakamura A, Shaw LM, Therapy, (2022) The performance of plasma amyloid beta measurements in identifying amyloid plaques in alzheimer’s disease: a literature review. Alzheimer’s Res 14:1–15 10.1186/s13195-022-01117-1 PMC9793600 36575454

[9] Alawode DO, Heslegrave AJ, Ashton NJ, Karikari TK, Simrén J, Montoliu-Gaya L, Pannee J, O’Connor A, Weston PS, Lantero-Rodriguez J (2021) Transitioning from cerebrospinal fluid to blood tests to facilitate diagnosis and disease monitoring in alzheimer’s disease. J Internal Med 290:583–601 34021943 10.1111/joim.13332 PMC8416781

[10] Schindler SE, Bollinger JG, Ovod V, Mawuenyega KG, Li Y, Gordon BA, Holtzman DM, Morris JC, Benzinger TL, Xiong C (2019) High-precision plasma β-amyloid 42/40 predicts current and future brain amyloidosis. Neurology 93:e1647–e1659 31371569 10.1212/WNL.0000000000008081 PMC6946467

[11] Bateman RJ, Munsell LY, Morris JC, Swarm R, Yarasheski KE, Holtzman DM (2006) Human amyloid-β synthesis and clearance rates as measured in cerebrospinal fluid in vivo. Nat Med 12:856–861 16799555 10.1038/nm1438 PMC2983090

[12] Keshavan A, Wellington H, Chen Z, Khatun A, Chapman M, Hart M, Cash DM, Coath W, Parker TD, Buchanan SM (2021) Concordance of csf measures of alzheimer’s pathology with amyloid pet status in a preclinical cohort: A comparison of lumipulse and established immunoassays. Alzheimer’s Dementia 13:e12131 33598527 10.1002/dad2.12131 PMC7867115

[13] Ovod V, Ramsey KN, Mawuenyega KG, Bollinger JG, Hicks T, Schneider T, Sullivan M, Paumier K, Holtzman DM, Morris JC (2017) Amyloid β concentrations and stable isotope labeling kinetics of human plasma specific to central nervous system amyloidosis. Alzheimer’s Dementia 13:841–849 28734653 10.1016/j.jalz.2017.06.2266 PMC5567785

[14] Klafki H-W, Morgado B, Wirths O, Jahn O, Bauer C, Esselmann H, Schuchhardt J, Wiltfang J (2022) Is plasma amyloid-β 1–42/1-40 a better biomarker for Alzheimer’s disease than aβx–42/x–40? Fluids Barriers CNS 19:96 36461122 10.1186/s12987-022-00390-4 PMC9719149

[15] Palmqvist S, Janelidze S, Stomrud E, Zetterberg H, Karl J, Zink K, Bittner T, Mattsson N, Eichenlaub U, Blennow K (2019) Performance of fully automated plasma assays as screening tests for Alzheimer disease–related β-amyloid status. JAMA Neurol 76:1060–1069 31233127 10.1001/jamaneurol.2019.1632 PMC6593637

[16] Verberk IM, Slot RE, Verfaillie SC, Heijst H, Prins ND, van Berckel BN, Scheltens P, Teunissen CE, van der Flier WM (2018) Plasma amyloid as prescreener for the earliest Alzheimer pathological changes. Ann Neurol 84:648–658 30196548 10.1002/ana.25334 PMC6282982

[17] Shahpasand-Kroner H, Klafki H-W, Bauer C, Schuchhardt J, Hüttenrauch M, Stazi M, Bouter C, Wirths O, Vogelgsang J, Wiltfang J (2018) A two-step immunoassay for the simultaneous assessment of aβ38, aβ40 and aβ42 in human blood plasma supports the aβ42/aβ40 ratio as a promising biomarker candidate of Alzheimer’s disease. Alzheimer’s Res 10:1–14 10.1186/s13195-018-0448-x PMC6286509 30526652

[18] Nakamura A, Kaneko N, Villemagne VL, Kato T, Doecke J, Doré V, Fowler C, Li Q-X, Martins R, Rowe C (2018) High performance plasma amyloid-β biomarkers for Alzheimer’s disease. Nature 554:249–254 29420472 10.1038/nature25456

[19] Janelidze S, Teunissen CE, Zetterberg H, Allué JA, Sarasa L, Eichenlaub U, Bittner T, Ovod V, Verberk IM, Toba K (2021) Head-to-head comparison of 8 plasma amyloid-β 42/40 assays in Alzheimer disease. JAMA Neurol 78:1375–1382 34542571 10.1001/jamaneurol.2021.3180 PMC8453354

[20] Verberk IM, Misdorp EO, Koelewijn J, Ball AJ, Blennow K, Dage JL, Fandos N, Hansson O, Hirtz C, Janelidze S (2022) Characterization of pre-analytical sample handling effects on a panel of Alzheimer’s disease–related blood-based biomarkers: Results from the standardization of Alzheimer’s blood biomarkers (sabb) working group. Alzheimers Dement 18:1484–1497 34845818 10.1002/alz.12510 PMC9148379

[21] Vogelgsang J, Shahpasand-Kroner H, Vogelgsang R, Streit F, Vukovich R, Wiltfang J (2018) Multiplex immunoassay measurement of amyloid-β 42 to amyloid-β 40 ratio in plasma discriminates between dementia due to Alzheimer’s disease and dementia not due to Alzheimer’s disease. Exp Brain Res 236:1241–1250 29480353 10.1007/s00221-018-5210-x

[22] Klafki H-W, Vogelgsang J, Manuilova E, Bauer C, Jethwa A, Esselmann H, Jahn-Brodmann A, Osterloh D, Lachmann I, Breitling B (2022) Diagnostic performance of automated plasma amyloid-β assays combined with pre-analytical immunoprecipitation. Alzheimer’s Res Therapy 14:1–12 10.1186/s13195-022-01071-y PMC9450259 36071505

[23] Mielke MM, Dage JL, Frank RD, Algeciras-Schimnich A, Knopman DS, Lowe VJ, Bu G, Vemuri P, Graff-Radford J, Jack CR Jr (2022) Performance of plasma phosphorylated tau 181 and 217 in the community. Nat Med 28:1398–1405 35618838 10.1038/s41591-022-01822-2 PMC9329262

[24] Janelidze S, Mattsson N, Palmqvist S, Smith R, Beach TG, Serrano GE, Chai X, Proctor NK, Eichenlaub U, Zetterberg H (2020) Plasma p-tau181 in alzheimer’s disease: relationship to other biomarkers, differential diagnosis, neuropathology and longitudinal progression to Alzheimer’s dementia. Nat Med 26:379–386 32123385 10.1038/s41591-020-0755-1

[25] Qu Y, Ma Y-H, Huang Y-Y, Ou Y-N, Shen X-N, Chen S-D, Dong Q, Tan L, Yu J-T (2021) Blood biomarkers for the diagnosis of amnestic mild cognitive impairment and Alzheimer’s disease: a systematic review and meta-analysis. Neurosci Biobehav Rev 128:479–486 34245759 10.1016/j.neubiorev.2021.07.007

[26] Martínez-Dubarbie F, Guerra-Ruiz A, López-García S, Lage C, Fernández-Matarrubia M, Infante J, Pozueta-Cantudo A, García-Martínez M, Corrales-Pardo A, Bravo M (2023) Accuracy of plasma aβ40, aβ42, and p-tau181 to detect csf alzheimer’s pathological changes in cognitively unimpaired subjects using the lumipulse automated platform. Alzheimer’s Res Therapy 15:1–11 10.1186/s13195-023-01319-1 PMC10544460 37784138

[27] Jack CR Jr et al (2018) NIA-AA research framework: toward a biological definition of Alzheimer’s disease. Alzheimer’s Dementia 14(4):535–562 29653606 10.1016/j.jalz.2018.02.018 PMC5958625

[28] Calero O, Hortigüela R, Bullido MJ, Calero M (2009) Apolipoprotein e genotyping method by real time pcr, a fast and cost-effective alternative to the taqman® and fret assays. J Neurosci Methods 183:238–240 19583979 10.1016/j.jneumeth.2009.06.033

[29] Efron B (1983) Estimating the error rate of a prediction rule: Improvement on cross-validation. J Am Stat Assoc 78:316–331

[30] Vandijck M, Degrieck R, Denoyette M, Delanote J, De Jonge M, De Decker B, Bastard NL, Vandenbroucke I (2022) Analytical performance of the lumipulse® g β-amyloid 1-40 plasma and lumipulse® g β-amyloid 1–42 plasma ruo assays. Alzheimers Dement 18:e068990

[31] Vandijck M, Dhont J, Dekeyser F, Bastard NL, Vandenbroucke I (2022) Analytical performance overview of the lumipulse g ptau 181 plasma ruo. Alzheimer’s Dementia 18:e064041

[32] Tijms BM, Willemse EA, Zwan MD, Mulder SD, Visser PJ, van Berckel BN, van der Flier WM, Scheltens P, Teunissen CE (2018) Unbiased approach to counteract upward drift in cerebrospinal fluid amyloid-β 1–42 analysis results. Clin Chem 64:576–585 29208658 10.1373/clinchem.2017.281055

